# Growth Differentiation Factor-15 and Clinical Outcomes in Japanese Patients With Ischemic Heart Disease

**DOI:** 10.1016/j.jacasi.2023.03.008

**Published:** 2023-05-09

**Authors:** Yuta Kobayashi, Yoichiro Otaki, Tetsu Watanabe, Shingo Tachibana, Junya Sato, Yuji Saito, Tomonori Aono, Jun Goto, Shigehiko Kato, Harutoshi Tamura, Satoshi Nishiyama, Takanori Arimoto, Hiroki Takahashi, Masafumi Watanabe

**Affiliations:** Department of Cardiology, Pulmonology, and Nephrology, Yamagata University School of Medicine, Yamagata, Japan

**Keywords:** clinical outcomes, GDF-15, high bleeding risk, ischemic heart disease

## Abstract

**Background:**

Despite a reduction in the rate of thrombotic events, ischemic heart disease (IHD) remains a key medical problem associated with high major bleeding and mortality in Asian patients with IHD. Growth differentiation factor (GDF)-15, a stress-response cytokine belonging to the transforming growth factor beta superfamily, is reportedly associated with poor clinical outcomes in Western patients with IHD. However, the clinical significance of GDF-15 in Asian patients with IHD has not yet been fully elucidated.

**Objectives:**

The aim of the present study was to examine the impact of serum GDF-15 on clinical outcomes in Japanese patients with IHD.

**Methods:**

Serum GDF-15 levels were evaluated in 632 consecutive patients with IHD. All patients were followed up for a median period of 2.8 years. The primary endpoint was the all-cause mortality rate. Secondary endpoints were major adverse cardiovascular events (MACE), heart failure (HF)–related rehospitalization, bleeding, and thrombotic events.

**Results:**

Serum GDF-15 levels were elevated in acute coronary syndrome, severe coronary artery disease, and the major Japanese version of the high bleeding risk criteria. Multivariate Cox proportional hazards regression analysis demonstrated that GDF-15 was an independent predictor of all-cause mortality, MACE, HF-related rehospitalizations, and bleeding events after adjusting for confounding risk factors but not for thrombotic events. Adding GDF-15 to risk factors significantly improved the net reclassification index and integrated discrimination improvement for all-cause deaths, MACE, HF-related rehospitalization, and bleeding events.

**Conclusions:**

Serum GDF-15 could be a feasible marker for major bleeding and adverse clinical outcomes in Japanese patients with IHD.

Ischemic heart disease (IHD) is a leading cause of death worldwide. Because of advances in revascularization and antithrombotic medicine, the thrombotic event rate has decreased significantly among patients with IHD who undergo revascularization.^1^ However, mortality in patients with IHD who undergo revascularization remains high. Therefore, early identification and risk stratification of patients at high risk for all-cause death are of critical importance.

Bleeding events in patients with IHD are associated with high mortality risk.^2^^,^^3^ Dual antiplatelet therapy is the standard of care for patients with IHD who undergo revascularization, which may increase the tendency for bleeding.^4^ Furthermore, Asian patients with IHD are characterized by high bleeding risk (HBR) compared with Caucasian patients with IHD.^5^ Therefore, an assessment of bleeding risk using the Japanese version of HBR (J-HBR) is recommended to determine the intensity and duration of antiplatelet and anticoagulation therapy according to the Japanese Circulation Society 2020 guidelines focused on antithrombotic therapy in patients with coronary artery disease.^6^ Some components of J-HBR, such as chronic kidney disease (CKD), anemia, heart failure (HF), atrial fibrillation (AF) and/or oral anticoagulation, and peripheral vascular disease (PVD), were also risk factors for thrombotic events. Therefore, a biomarker is required to discriminate between thrombotic and bleeding risks and to predict subsequent all-cause mortality.

Growth differentiation factor (GDF)-15 is a member of the transforming growth factor-beta superfamily. Although GDF-15 is appreciably expressed in the liver and placenta at baseline, circulating GDF-15 levels are generally low in healthy individuals. GDF-15 is induced by several factors, such as oxidative stress, inflammation, and mechanical stress in various tissues, and is released into circulation.^7^^,^^8^ As circulating GDF-15 is reportedly elevated in advanced age, cancer, and cardiovascular disease,^9^ it is considered a stress-response cytokine.

Accumulating evidence has demonstrated that GDF-15 can help predict mortality, major adverse cardiovascular events (MACE), and HF hospitalizations in patients with IHD.^10^, ^11^, ^12^, ^13^ Moreover, GDF-15 is associated with major bleeding in patients with AF across different geographic areas, including Asia.^14^^,^^15^

GDF-15 measurement is used clinically for risk stratification of clinical outcomes in patients with acute coronary syndrome (ACS) and chronic HF and assessment of bleeding risk in patients with AF in Western countries. However, the clinical usefulness of serum GDF-15 levels in Asian populations has not yet been fully elucidated. Notably, it remains undetermined whether GDF-15 could identify Asian patients with IHD at high risk for bleeding events. Considering the HBR in Asian patients, we focused on an association of serum GDF-15 with bleeding events and all-cause death in Asian patients with IHD. The aims of the present study were to: 1) examine the association between serum GDF-15 levels and coronary artery disease severity and J-HBR; and 2) examine the impact of serum GDF-15 on clinical outcomes such as mortality, MACE, HF-related rehospitalization, bleeding events, and thrombotic events in Japanese patients with IHD.

## Methods

### Ethics statement

All procedures involving participants were undertaken in accordance with the ethical, institutional, and/or national research committee guidelines of the centers at which the studies were conducted (Yamagata University, 2021-298), and all procedures complied with the 1964 Declaration of Helsinki and its later amendments or comparable ethical standards. The study was approved by the institutional ethics committee of Yamagata University School of Medicine.

### Study subjects

The study subjects were 632 patients with IHD who were admitted to Yamagata University Hospital for treatment between 2012 and 2018. The diagnoses of ACS and chronic coronary syndrome (CCS) were based on fourth universal definition of myocardial infarction and 2019 European Society of Cardiology guidelines, respectively.^16^^,^^17^ Invasive treatments, such as percutaneous coronary intervention (PCI) and/or coronary artery bypass graft surgery, were determined by expert cardiologists and cardiac surgeons according to the guidelines.^18^^,^^19^

Clinical data such as age, sex, hypertension, diabetes mellitus, dyslipidemia, smoking status, medical history, and medication use at discharge were obtained from the patients’ medical records and interviews.

### Serum GDF-15 level measurement and ABC-AF-bleeding score calculation

Blood samples were collected in the early morning within 24 hours of hospital admission. The samples were centrifuged at 3,000 rpm for 15 minutes at 4 °C, and the obtained serum was stored at −80 °C until analysis. The GDF-15 level assay was performed using an electrochemiluminescent immunoassay (Roche Diagnostics). Brain natriuretic peptide (BNP) levels were evaluated using a chemiluminescent enzyme immunoassay. Troponin T (TnT) and high sensitivity C-reactive protein (hsCRP) levels were evaluated using the electrochemiluminescent immunoassay and latex agglutination methods, respectively. Hemoglobin levels were measured simultaneously. The ABC-AF-bleeding score is composed of age, TnT, hemoglobin, GDF-15, and previous bleeding.^15^ The ABC-AF-bleeding score was evaluated using an online calculator.

### Cardiovascular risk factors

Hypertension was defined as systolic blood pressure ≥140 mm Hg, diastolic blood pressure ≥90 mm Hg, or antihypertensive medication use. Diabetes mellitus was defined as a fasting blood glucose level ≥126 mg/dL, glycated hemoglobin ≥6.5%, or antidiabetic medication use. Dyslipidemia was defined as high-density lipoprotein cholesterol <40 mg/dL, low-density lipoprotein cholesterol ≥140 mg/dL, triglyceride ≥150 mg/dL, or use of lipid-lowering medication.

### J-HBR

Bleeding risk was assessed using the J-HBR criteria according to the Japanese Circulation Society 2020 guideline recommendations.^6^ The primary criteria for J-HBR include low body weight (<55 kg in men, <50 kg in women), severe CKD (estimated glomerular filtration rate <30 mL/min/1.73 m^2^ or hemodialysis), moderate to severe anemia (hemoglobin <11 g/dL), HF, anticipated use of long-term oral anticoagulation, PVD, history of nontraumatic bleeding events, history of ischemic stroke or intracranial hemorrhage, thrombocytopenia (platelet count <100 × 10^9^/L), active malignancy, liver cirrhosis with portal hypertension, chronic bleeding diatheses, nondeferrable major surgery on dual antiplatelet therapy, and recent major surgery or major trauma within 30 days before PCI.

### Endpoints and follow-up period

All patients were prospectively followed up by telephone or by reviewing medical records twice a year for a median period of 2.8 years (IQR: 0.9-5.0 years). The primary endpoint was the all-cause mortality rate. The secondary endpoints were MACE, HF-related rehospitalization, bleeding, and thrombotic events. MACE includes ACS, stroke, cerebral hemorrhage, HF-related rehospitalization, fatal arrhythmias, and cardiac death, defined as death due to progressive ACS, HF, or sudden cardiac death. HF-related rehospitalization was defined as hospitalization for HF progression. Bleeding events were defined as Bleeding Academic Research Consortium type 3 or 5. Thrombotic events were defined as ACS, stroke, and peripheral vascular thrombosis.

### Statistical analysis

The normality of continuous variables was confirmed using the Shapiro-Wilk test. Results are expressed as mean ± SD for continuous variables and as number (percentage) for categorical variables. Skewed values are presented as median (IQR). Student’s *t*-test and the chi-square test were used to compare continuous and categorical variables, respectively. Differences in age, hemoglobin, and estimated glomerular filtration rate among the groups were analyzed using analysis of variance with Tukey’s post hoc test. Differences in TnT, BNP, hsCRP, and ABC-AF-bleeding score among groups were analyzed using the Steel-Dwass test.^20^ Survival curves were plotted using the Kaplan-Meier method and compared using the log-rank test. Because of the skewed distributions of TnT, BNP, hsCRP, and GDF-15 values, we checked supreme tests for linear form. Thus, we used log-transformed TnT, BNP, hsCRP, and GDF-15 values for the Cox proportional hazards regression analysis. Cox proportional hazards analysis was used to determine independent predictors of the primary and secondary endpoints. Multicollinearity was assessed using the variance inflation factor. Significant predictors selected in the univariate Cox proportional hazards regression analysis for each clinical outcome were entered into the multivariate analysis for all-cause mortality, MACE, HF-related rehospitalization, and thrombotic events. The proportionality assumption in the Cox model was assessed using the Schoenfeld residuals test. As BNP was used for the diagnosis of HF in the present study, there was a close relationship between BNP and HF. BNP is also a prognostic marker for IHD, so we entered BNP into multivariate analysis, but not HF. The major components of the J-HBR criteria were entered into the multivariate analysis for bleeding events. Thus, we entered HF into the multivariate analysis for bleeding events, but not BNP. As the definition of anemia differs by sex, we used hemoglobin in the Cox proportional hazards model. Receiver-operating characteristic curves for all-cause mortality, MACE, HF-related rehospitalization, and bleeding events were plotted and used to measure the predictive accuracy of serum GDF-15 level for these clinical outcomes. We calculated the net reclassification index (NRI) and integrated discrimination improvement (IDI) to measure the incremental value of serum GDF-15 in classifying patients at high or low risk for all-cause mortality, MACE, HF-related rehospitalizations, and bleeding events. Statistical significance was set at a *P* value of <0.05. All statistical analyses were performed using JMP version 14.2 (SAS Institute) and EZR (Saitama Medical Center, Jichi Medical University).

## Results

### Baseline characteristics of all patients with IHD and comparisons of clinical characteristics among the GDF-15 tertiles

The baseline characteristics of the 632 patients are summarized in Table 1. There were 484 patients (76.6%) with ACS and 148 (23.4%) with CCS. Significant stenosis in left main trunk lesions and 3-vessel diseases were observed in 53 (8.5%) and 147 (23.6%) patients, respectively. Hypertension, diabetes mellitus, dyslipidemia, and current smoking were identified in 480 (76%), 246 (39%), 371 (58.7%), and 400 (63.3%) patients, respectively. The median serum GDF-15 level was 1,991 pg/mL. PCI and coronary artery bypass graft surgery were performed in 565 (89.4%) and 41 (6.5%) patients with IHD, respectively, indicating that up to 94.9% of the patients underwent revascularization in this study. Major criteria for J-HBR, such as low body weight, severe CKD, moderate to severe anemia, HF, anticipated use of long-term oral anticoagulation, and PVD, were identified in 156 (24.7%), 97 (15.3%), 78 (12.3%), 160 (25.3%), 97 (15.3%), and 68 (10.8%) patients, respectively.

**Table 1:** Baseline Characteristics and Comparisons of Clinical Characteristics Among 3 Groups on the Basis of GDF-15 Level

	All Subjects (N = 632)	First Tertile (<1,509 pg/mL) (n = 211)	Second Tertile (1,509-2,604 pg/mL) (n = 211)	Third Tertile (>2,604 pg/mL) (n = 210)	*P* Value
Age, y	69.5 ± 11.5	61.8 ± 10.4	71.0 ± 10.0^a^	75.6 ± 9.5^a^^,^^b^	<0.0001
Men	479 (75.8)	170 (80.6)	159 (75.4)	150 (71.4)	0.0896
ACS/CCS	484/148	154/57	149/62	181/29	0.0003
LMT lesion	53 (8.5)	11 (5.2)	15 (7.2)	27 (13.3)	0.0228
3-vessel disease	147 (23.6)	33 (15.6)	49 (23.4)	65 (32.0)	0.0005
Hypertension	480 (76.0)	137 (64.9)	170 (80.6)	173 (82.4)	<0.0001
Diabetes mellitus	246 (39.0)	60 (28.0)	91 (43.3)	95 (45.2)	0.0006
Dyslipidemia	371 (58.7)	131 (62.1)	121 (57.4)	119 (56.7)	0.4688
Smoking	400 (63.3)	148 (70.1)	127 (60.2)	125 (59.5)	0.0404
Prior myocardial infarction	66 (10.4)	19 (9.0)	28 (13.3)	19 (9.1)	0.2583
Prior revascularization	111 (17.6)	38 (18.0)	43 (20.4)	30 (14.3)	0.2538
Prior atrial fibrillation	70 (11.1)	13 (6.2)	22 (10.4)	35 (16.7)	0.0026
Prior heart failure	35 (5.5)	4 (1.9)	12 (5.7)	19 (9.1)	0.0058
Prior bleeding	24 (3.8)	5 (2.4)	7 (3.3)	12 (5.7)	0.1806
Prior stroke	75 (11.9)	12 (5.7)	29 (13.7)	34 (16.2)	0.0023
Biochemical data					
eGFR, mL/min/1.73 m^2^	69.3 ± 25.1	80.7 ± 19.4	71.9 ± 19.1^a^	55.2 ± 28.5^a^^,^^b^	<0.0001
Hemoglobin, g/dL	13.3 ± 2.1	14.1 ± 1.7	13.5 ± 1.8^a^	12.5 ± 2.3^a^^,^^b^	<0.0001
Troponin T, pg/mL	1.24 (0.10-4.41)	0.69 (0.04-3.19)	0.72 (0.03-3.11)	2.61 (0.64-5.71)^c^^,^^d^	<0.0001
BNP, pg/mL	62.8 (23.1-205.7)	29.5 (16.1-71.2)	52.7 (22.0-170.7)^c^	166.8 (63.8-481.6)^c^^,^^d^	<0.0001
hsCRP, μg/mL	0.59 (0.12-2.67)	0.27 (0.07-1.58)	0.35 (0.08-2.07)	1.42 (0.45-5.14)^c^^,^^d^	<0.0001
GDF-15, pg/mL	1,991 (1,289-3,060)				
ABC-AF-bleeding score	4.96 (2.74-8.17)	2.89 (1.33-4.01)	5.01 (2.95-6.36)^c^	9.96 (6.77-13.57)^c^^,^^d^	<0.0001
Medications					
ACE inhibitors and/or ARBs	516 (81.6)	169 (80.1)	176 (83.4)	171 (81.4)	0.6740
β-blockers	378 (59.8)	123 (58.3)	121 (57.4)	134 (63.8)	0.3421
MRAs	53 (8.4)	9 (4.3)	11 (5.2)	33 (15.7)	<0.0001
Statins	553 (87.8)	198 (94.3)	191 (91.0)	164 (78.1)	<0.0001
Aspirin	596 (94.3)	204 (96.7)	201 (95.3)	191 (91.0)	0.0306
P2Y_12_ receptor antagonists	540 (85.4)	192 (90.5)	181 (85.8)	167 (79.5)	0.0038
Revascularization	600 (94.9)	205 (97.2)	202 (95.7)	193 (91.9)	0.0396
PCI	565 (89.4)	198 (93.8)	191 (90.5)	176 (83.8)	0.0030
CABG	41 (6.5)	9 (4.3)	14 (6.6)	18 (8.6)	0.1900
Japanese version of HBR criteria					
Low body weight	156 (24.7)	35 (16.6)	57 (27.3)	64 (31.7)	0.0013
Severe CKD	97 (15.3)	6 (2.8)	21 (10.0)	70 (33.3)	<0.0001
Moderate to severe anemia	78 (12.3)	6 (2.8)	18 (8.5)	54 (25.7)	<0.0001
Heart failure	160 (25.3)	20 (9.5)	48 (22.8)	92 (43.8)	<0.0001
Anticipated use of long-term oral anticoagulation	97 (15.3)	21 (10.0)	32 (15.2)	44 (21.0)	0.0074
Peripheral vascular disease	68 (10.8)	11 (5.2)	23 (10.9)	34 (16.2)	0.0014
History of nontraumatic bleeding events	10 (1.6)	2 (1.0)	1 (0.5)	7 (3.3)	0.0419
Previous ischemic stroke or ICH	13 (2.1)	3 (1.4)	5 (2.4)	5 (2.4)	0.7282
Thrombocytopenia (platelet count <100 × 10^9^/L)	8 (1.3)	3 (1.4)	0 (0.0)	5 (2.4)	0.0891
Active malignancy	27 (4.3)	4 (1.9)	7 (3.3)	16 (7.6)	0.0104
Liver cirrhosis with portal hypertension	4 (0.6)	0 (0.0)	1 (0.5)	3 (1.4)	0.1701
Chronic bleeding diatheses	0 (0.0)	0 (0.0)	0 (0.0)	0 (0.0)	
Nondeferrable major surgery on DAPT	27 (4.3)	6 (2.8)	11 (5.2)	10 (4.8)	0.4420
Recent major surgery or major trauma within 30 d before PCI	4 (0.6)	0 (0.0)	3 (1.4)	1 (0.5)	0.1726

Values are mean ± SD, n (%), n, or median (IQR).

ACE = angiotensin-converting enzyme inhibitor; ACS = acute coronary syndrome; ARB = angiotensin receptor blocker; BNP = brain natriuretic peptide; CABG = coronary artery bypass graft; CCS = chronic coronary syndrome; CKD = chronic kidney disease; DAPT = dual antiplatelet therapy; eGFR = estimated glomerular filtration rate; GDF= growth differentiation factor; HBR = high bleeding risk; hsCRP = high-sensitivity C-reactive protein; ICH = intracranial hemorrhage; LMT = left main trunk; MRA = mineral corticoid receptor antagonist; PCI = percutaneous coronary intervention.

a *P* < 0.05 vs first tertile.

b *P* < 0.05 vs second tertile by analysis of variance with Tukey’s post hoc test.

c *P* < 0.05 vs first tertile.

d *P* < 0.05 vs second tertile by Steel-Dwass test.

**Figure 1:**
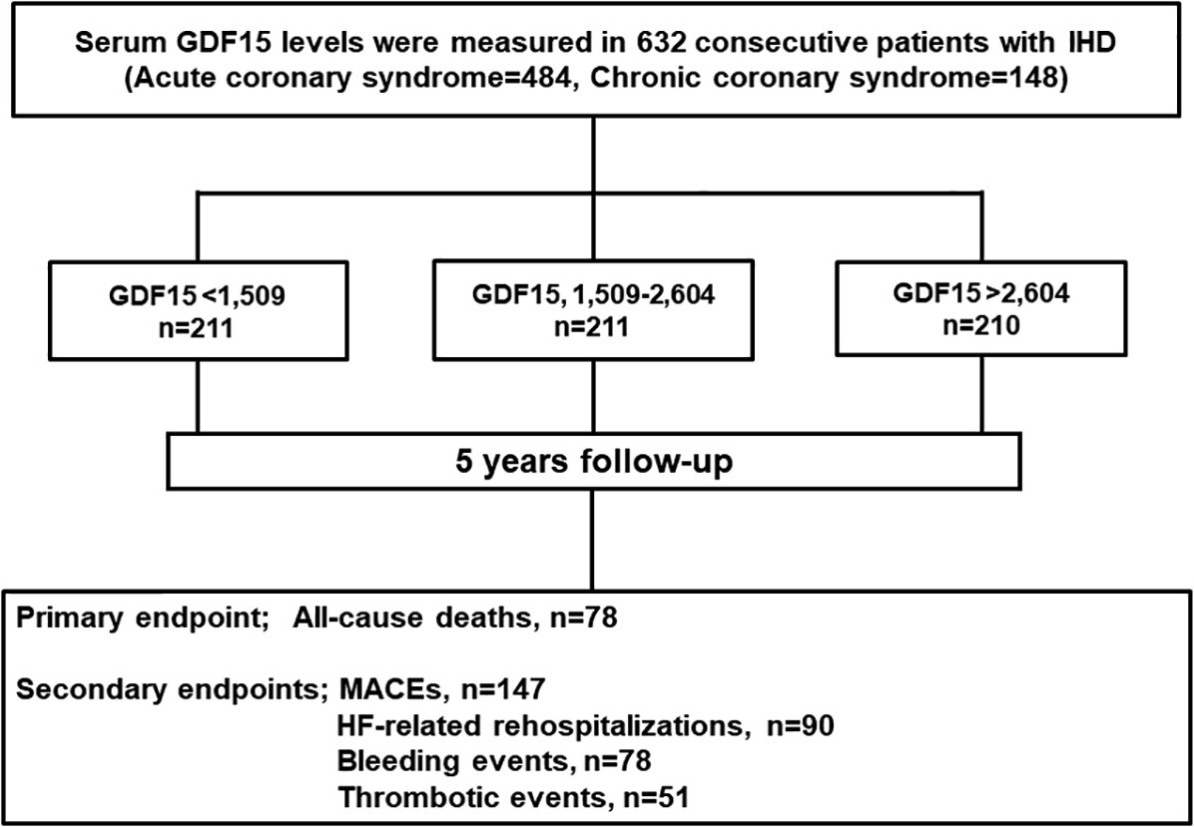
Study Flowchart Flowchart of the present study. GDF= growth differentiation factor; HF = heart failure; IHD = ischemic heart disease; MACE = major adverse cardiovascular event(s).

As shown in Figure 1, all patients were divided into 3 groups according to serum GDF-15 tertiles: first tertile group, GDF-15 <1,509 pg/mL (n = 211); second tertile group, GDF-15 1,509 to 2,604 pg/mL (n = 211); and third tertile group, GDF-15 >2,604 pg/mL (n = 210). The third tertile group was older and had higher prevalence rates of ACS, left main trunk lesions, 3-vessel disease, hypertension, diabetes mellitus, prior AF, prior HF, and prior stroke compared with the other groups. Additionally, the third tertile group showed lower estimated glomerular filtration rate and hemoglobin levels, higher TnT, BNP, hsCRP levels, and higher ABC-AF-bleeding scores than the other groups. The third tertile group had higher prevalence rates of low body weight, severe CKD, moderate to severe anemia, HF, anticipated use of long-term oral anticoagulation, PVD, history of nontraumatic bleeding events, and active malignancy than the other groups (Table 1).

### Association of serum GDF-15 with coronary artery disease severity and J-HBR criteria

**Figure 2:**
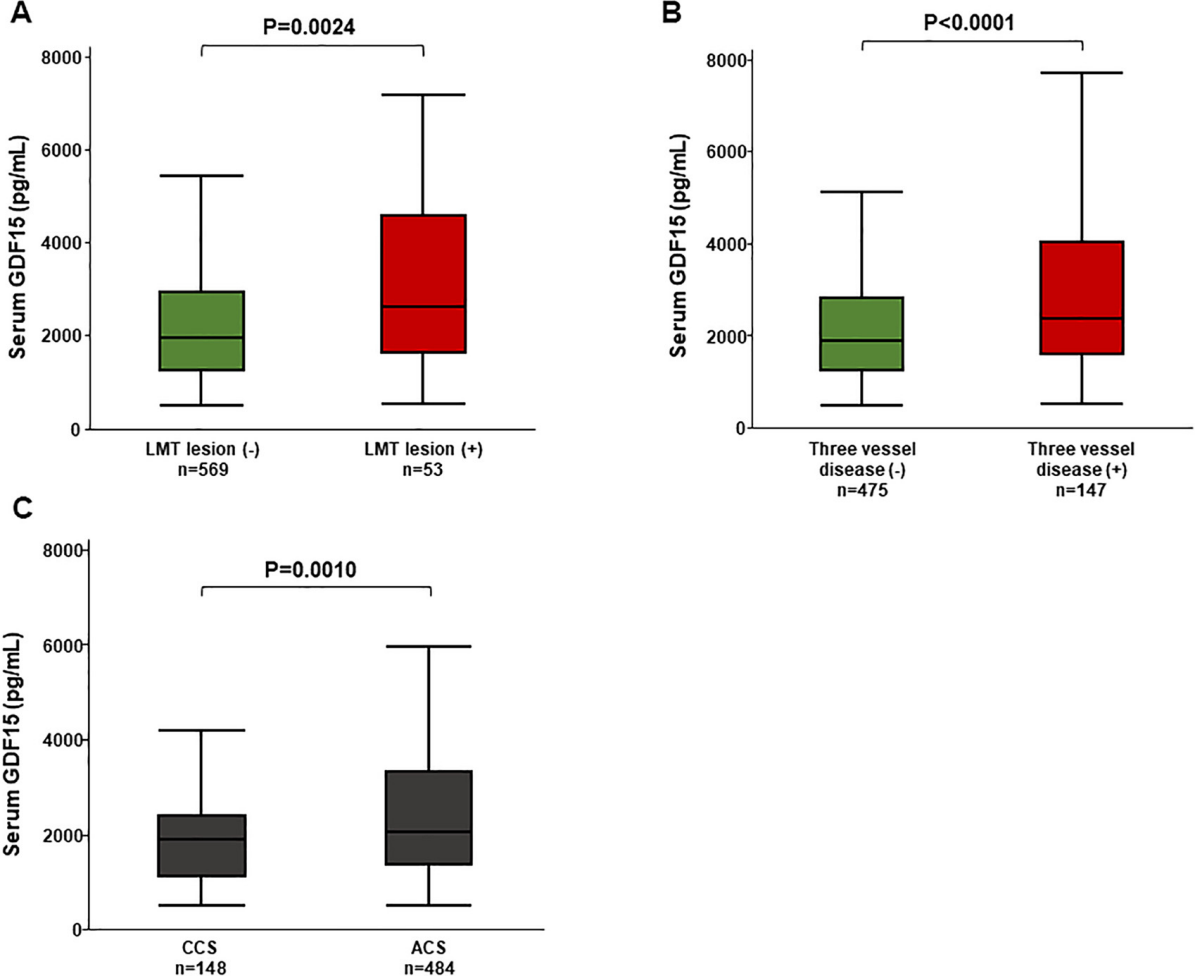

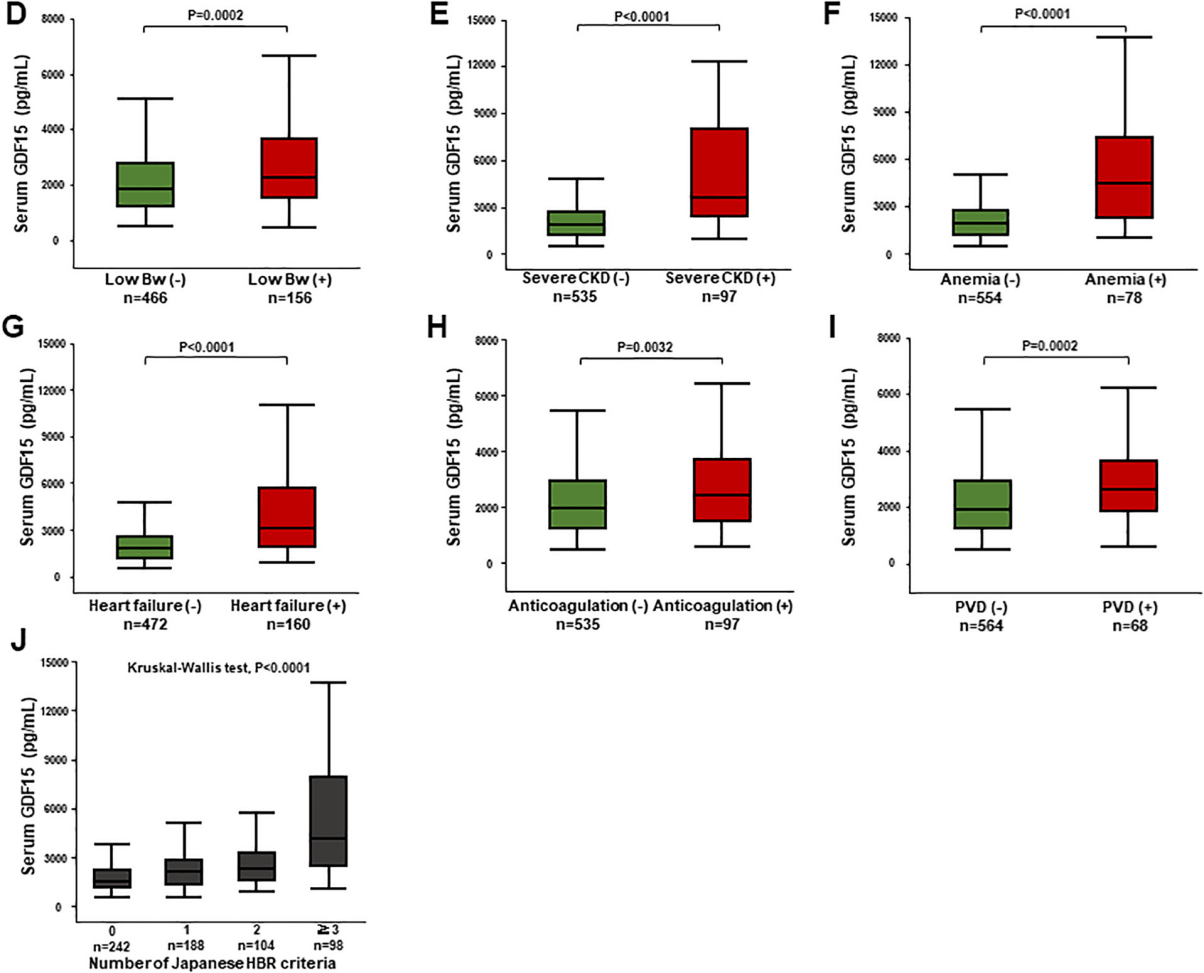
Associations of Serum GDF-15 With IHD Severity and J-HBR Associations of serum GDF-15 level with left main trunk (LMT) lesion **(A)**, 3-vessel disease **(B)**, and diagnosis of IHD **(C)**. Associations of serum GDF-15 level with low body weight (Bw) **(D)**, severe chronic kidney disease (CKD) **(E)**, moderate to severe anemia **(F)**, heart failure **(G)**, anticipated use of long-term oral anticoagulation **(H)**, and peripheral vascular disease (PVD) **(I)**. Association between serum GDF-15 level and an accumulation of the Japanese version of high bleeding risk (J-HBR). ACS = acute coronary syndrome; CCS = chronic coronary syndrome; HBR = high bleeding risk; other abbreviations as in Figure 1.

Serum GDF-15 levels were elevated in patients with severe coronary artery disease, such as left main trunk lesions and 3-vessel disease (Figures 2A and 2B). Furthermore, serum GDF-15 levels were higher in patients with ACS than in those with CCS (Figure 2C). As shown in Figures 2D to 2I, serum GDF-15 levels were significantly elevated in patients with low body weight, severe CKD, moderate to severe anemia, HF, anticipated use of long-term oral anticoagulation, and PVD. Notably, serum GDF-15 levels increased according to the J-HBR criteria (Figure 2J, Central Illustration).

**Central Illustration:**
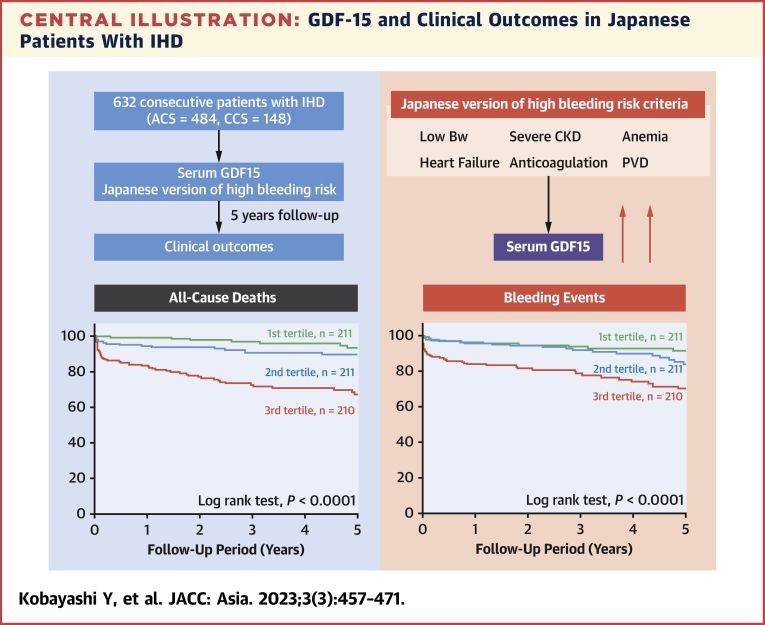
GDF-15 and Clinical Outcomes in Japanese Patients With IHD Serum growth differentiation factor (GDF)-15 elevation was related to the Japanese version of high bleeding risk. GDF-15 could stratify patients with ischemic heart disease (IHD) at high risk for all-cause death and major bleeding events. ACS = acute coronary syndrome; Bw = body weight; CCS = chronic coronary syndrome; CKD = chronic kidney disease; PVD = peripheral vascular disease.

### Serum GDF-15 levels and primary endpoint

**Figure 3:**
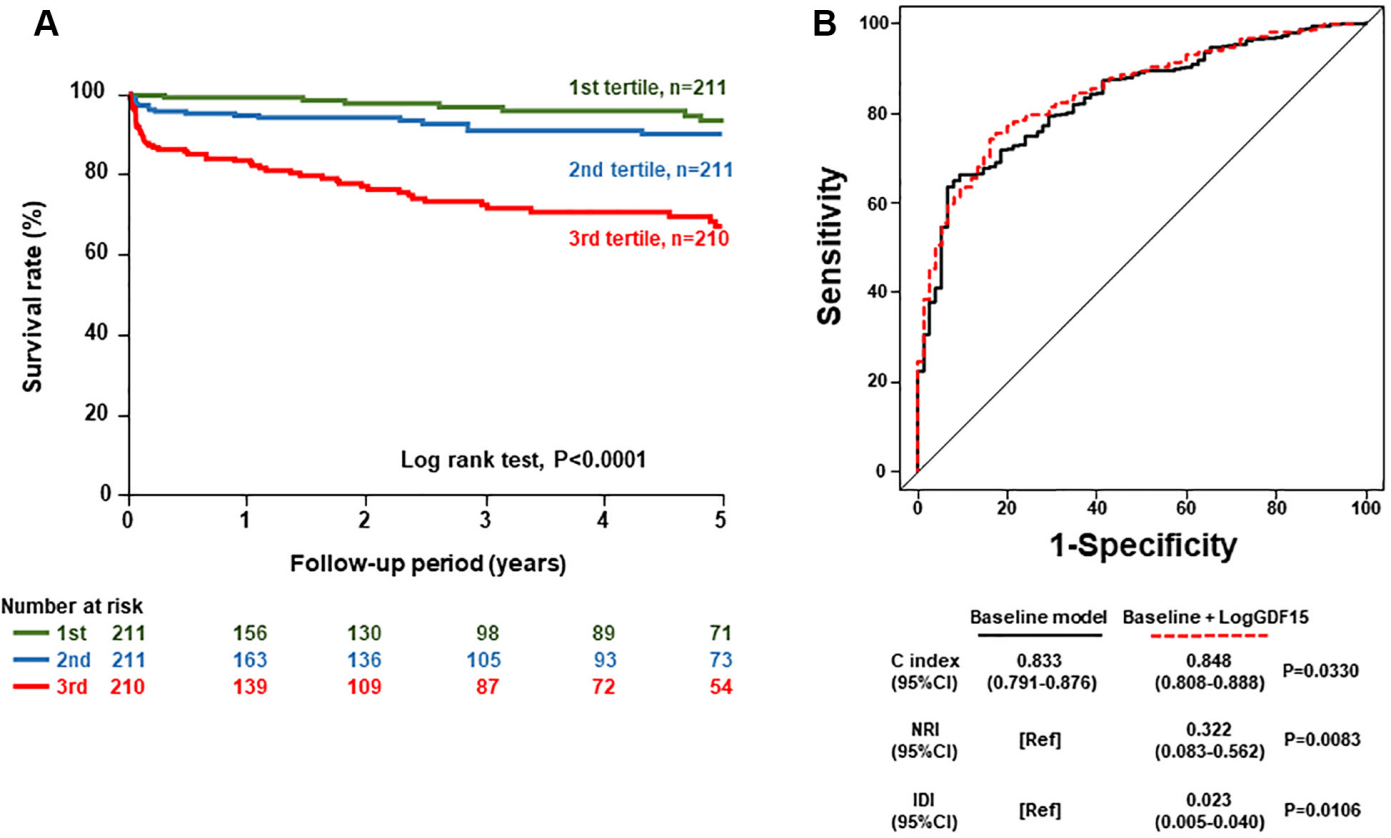
Prediction Capacity of Serum GDF-15 for Primary Outcome **(A)** Kaplan-Meier analysis for all-cause mortality among serum growth differentiation factor (GDF)-15 tertiles. **(B)** Receiver-operating characteristic curve for all-cause mortality. Comparison of C index, net reclassification improvement (NRI), and integrated discrimination improvement (IDI) for all-cause mortality between baseline model with and without serum GDF-15. The baseline model includes age, sex, hemoglobin, troponin T, brain natriuretic peptide, high-sensitivity C-reactive protein, revascularization, and severe chronic kidney disease. Ref = reference.

During the follow-up period, 78 all-cause deaths occurred in this study. As shown in Figure 3A, Kaplan-Meier analysis revealed that the highest tertile group had the greatest risk for all-cause mortality among the 3 groups (Central Illustration). Multivariate Cox proportional hazards regression analysis demonstrated that serum GDF-15 level was an independent predictor of all-cause mortality after adjusting for age, sex, hemoglobin, log TnT, log BNP, log hsCRP, revascularization, and severe CKD (HR: 1.42; 95% CI: 1.11-1.79; *P* = 0.0038) (Table 2). To examine whether the model fit and discrimination improved when serum GDF-15 was added to predictors selected in univariate Cox proportional hazards regression analysis, we evaluated improvements in the C index, NRI, and IDI. Serum GDF-15 significantly improved the C index, with significant NRI and IDI (Figure 3B).

**Table 2:** Univariate and Multivariate Cox Proportional Hazards Analyses for All-Cause Mortality in Patients With Ischemic Heart Disease

	Univariate Analysis			Multivariate Analysis		
	HR	95% CI	*P* Value	HR	95% CI	*P* Value
Age^a^	2.80	2.11-3.76	<0.0001	1.78	1.32-2.43	0.0002
Men vs women	0.42	0.27-0.66	0.0002	0.87	0.52-1.43	0.5722
ACS vs CCS	1.57	0.89-2.76	0.1178			
Hypertension	1.85	0.98-3.50	0.0591			
Diabetes mellitus	0.95	0.60-1.50	0.8310			
Hb^a^	0.45	0.37-0.55	<0.0001	0.81	0.62-1.06	0.1303
Log TnT^a^	1.39	1.09-1.79	0.0095	1.11	0.81-1.55	0.5144
Log BNP^a^	2.82	2.24-3.56	<0.0001	1.99	1.46-2.75	<0.0001
Log hsCRP^a^	1.54	1.21-1.95	0.0004	1.05	0.77-1.44	0.7787
Log GDF-15^a^	2.06	1.75-2.40	<0.0001	1.42	1.11-1.79	0.0038
Revascularization	0.29	0.15-0.58	0.0005	0.76	0.35-1.66	0.4946
Low body weight	1.46	0.88-2.42	0.1460			
Severe CKD	2.02	1.23-3.34	0.0058	0.59	0.34-1.02	0.0589
Moderate to severe anemia	4.00	2.48-6.44	<0.0001			
Heart failure	5.18	3.30-8.14	<0.0001			
Anticipated use of long-term oral anticoagulation	0.64	0.31-1.33	0.2345			
PVD	1.01	0.51-2.03	0.9724			

Hb = hemoglobin; PVD = peripheral vascular disease; TnT = troponin T; other abbreviations as in Table 1.

a Per 1 SD increase.

### Serum GDF-15 levels and secondary endpoints

**Figure 4:**
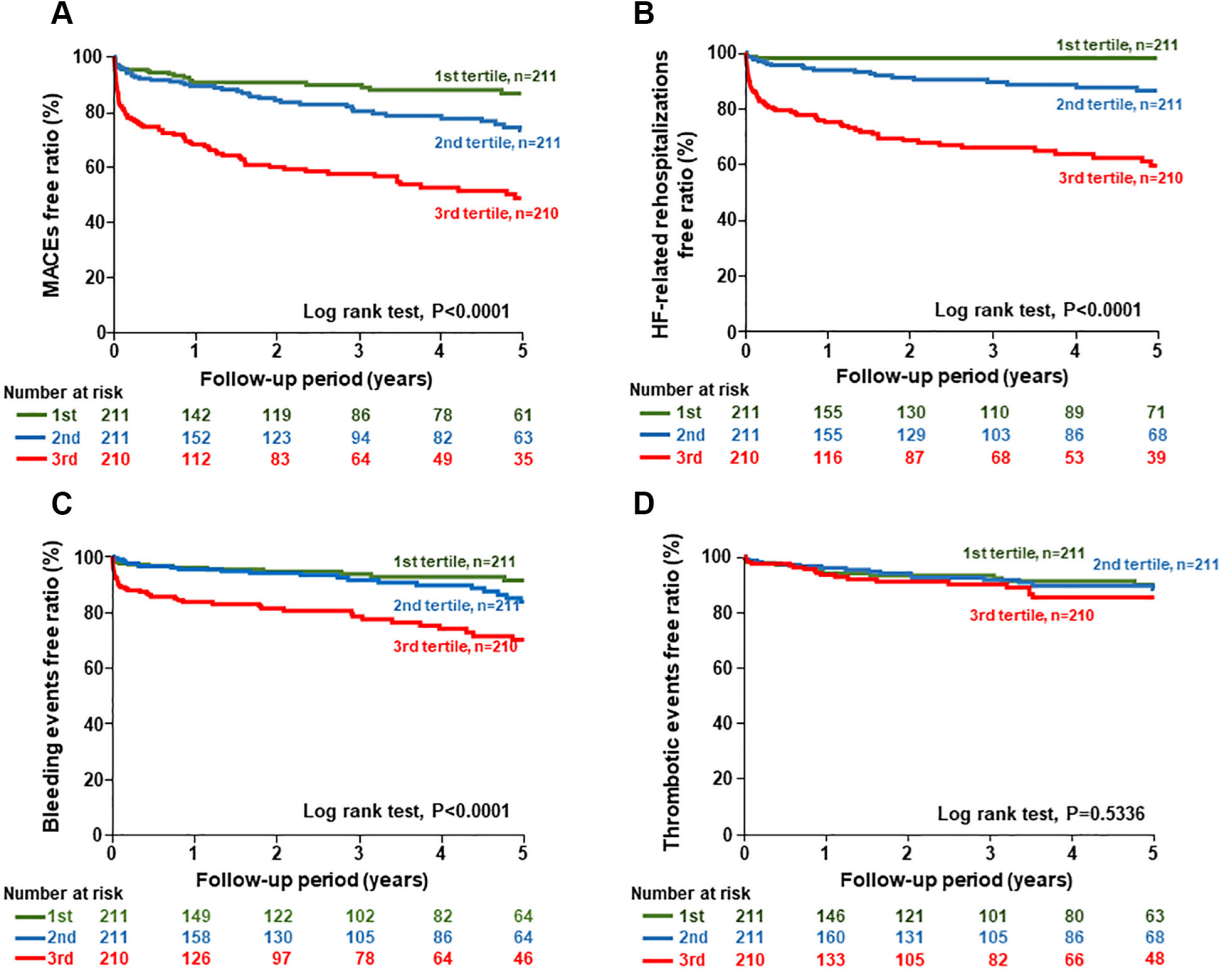
Different Risk for Secondary Outcomes Among Serum Growth Differentiation Factor-15 Tertiles Kaplan-Meier analysis for major adverse cardiovascular events (MACE) **(A)**, heart failure (HF)-related rehospitalization **(B)**, bleeding events **(C)**, and thrombotic events **(D)** among serum growth differentiation factor-15 tertiles.

During the follow-up period, 148 MACE, 90 HF-related hospitalizations, 78 bleeding events, and 51 thrombotic events occurred. As shown in Figure 4, Kaplan-Meier analysis revealed that the highest tertile group had the greatest risk for MACE, HF-related hospitalization, and bleeding events among the 3 groups (Central Illustration). In contrast, the thrombotic event rate was not significantly different among the serum GDF-15 tertile groups.

Multivariate Cox proportional hazards regression analysis demonstrated that serum GDF-15 level was an independent predictor for MACE, HF-related hospitalization, and bleeding events after adjustment for confounding risk factors (Table 3). Similarly, ABC-AF-bleeding score was an independent predictor for bleeding events. However, a significant association was not maintained in the multivariate Cox proportional hazards regression analysis for thrombotic events.

**Table 3:** Univariate and Multivariate Cox Proportional Hazards Analyses for Secondary Endpoints in Patients With Ischemic Heart Disease

	Univariate Analysis			Multivariate Analysis		
	HR	95% CI	*P* Value	HR	95% CI	*P* Value
MACE						
Age^a^	1.54	1.28-1.85	<0.0001	0.99	0.81-1.22	0.9379
Men vs women	0.55	0.39-0.78	0.0007	0.69	0.47-1.01	0.0540
ACS vs CCS	1.39	0.94-2.06	0.1019			
Hypertension	1.61	1.04-2.49	0.0336	0.96	0.61-1.50	0.8455
Diabetes mellitus	1.33	0.96-1.84	0.0813			
Hb^a^	0.61	0.52-0.71	<0.0001	0.97	0.80-1.17	0.7318
Log TnT^a^	1.14	1.13-1.60	0.0011	1.12	0.90-1.41	0.3118
Log BNP^a^	2.43	2.06-2.88	<0.0001	1.87	1.51-2.34	<0.0001
Log hsCRP^a^	1.43	1.20-1.69	<0.0001	1.03	0.83-1.29	0.7950
Log GDF-15^a^	2.02	1.76-2.30	<0.0001	1.43	1.17-1.74	0.0003
Revascularization	0.52	0.27-0.99	0.0473	1.09	0.55-2.16	0.8063
Low body weight	1.40	0.98-2.01	0.0657			
Severe CKD	2.83	2.00-4.00	<0.0001	1.08	0.71-1.66	0.7089
Moderate to severe anemia	2.77	1.90-4.06	<0.0001			
Heart failure	4.05	2.92-5.60	<0.0001			
Anticipated use of long-term oral anticoagulation	1.85	1.26-2.71	0.0017	1.38	0.93-2.06	0.1082
PVD	1.67	1.09-2.57	0.0194	1.47	0.93-2.32	0.0997
HF-related rehospitalization						
Age^a^	1.90	1.49-2.45	<0.0001	1.05	0.79-1.41	0.7323
Men vs women	0.48	0.31-0.73	0.0007	0.66	0.40-1.10	0.1115
ACS vs CCS	1.47	0.88-2.47	0.1440			
Hypertension	1.73	0.98-3.05	0.0604			
Diabetes mellitus	1.50	0.99-2.67	0.0550			
Hb^a^	0.57	0.86-1.60	<0.0001	0.87	0.67-1.12	0.2907
Log TnT^a^	1.51	1.20-1.93	0.0007	1.25	0.93-1.71	0.1474
Log BNP^a^	3.38	2.72-4.22	<0.0001	2.23	1.67-3.01	<0.0001
Log hsCRP^a^	1.53	1.23-1.91	0.0002	0.85	0.64-1.14	0.2752
Log GDF-15^a^	2.51	2.13-2.95	<0.0001	1.61	1.26-2.05	0.0001
Revascularization	0.57	0.25-1.30	0.1798			
Low body weight	1.82	1.16-2.85	0.0089	0.88	0.53-1.47	0.6301
Severe CKD	4.25	2.79-6.47	<0.0001	1.18	0.69-2.01	0.5393
Moderate to severe anemia	4.28	2.74-6.67	<0.0001			
Heart failure	7.53	4.86-11.65	<0.0001			
Anticipated use of long-term oral anticoagulation	2.41	1.53-3.81	0.0002	2.00	1.24-3.21	0.0044
PVD	1.23	0.67-2.25	0.5127			
Bleeding events						
Age^a^	1.68	1.30-2.18	<0.0001	1.26	0.94-1.70	0.1273
Men vs women	0.66	0.40-1.07	0.0931			
ACS vs CCS	1.08	0.65-1.81	0.7554			
Hypertension	1.85	0.98-3.49	0.0596			
Diabetes mellitus	0.83	0.53-1.32	0.4349			
Prior bleeding	1.32	0.48-3.60	0.5937			
Hb^a^	0.57	0.46-0.71	<0.0001	0.74	0.56-0.96	0.0240
Log TnT^a^	1.14	0.91-1.44	0.2580			
Log BNP	1.80	1.44-2.26	<0.0001			
Log hsCRP	1.35	1.07-1.70	0.0119			
Log GDF-15^a^	1.78	1.48-2.11	<0.0001	1.57	1.24-1.96	0.0001
Low body weight	1.72	1.08-2.75	0.0229	0.89	0.53-1.50	0.6691
Severe CKD	1.80	1.07-3.02	0.0257	0.66	0.36-1.20	0.1732
Moderate to severe anemia	3.14	1.89-5.23	<0.0001			
Heart failure	2.72	1.74-4.28	<0.0001	1.49	0.87-2.54	0.1463
Anticipated use of long-term oral anticoagulation	1.58	0.91-2.73	0.1050	1.33	0.76-2.33	0.3254
PVD	1.43	0.77-2.64	0.2589	1.14	0.61-2.13	0.6797
Thrombotic events						
Age^a^	1.19	0.89-1.62	0.2415			
Men vs women	0.67	0.37-1.22	0.1888			
ACS vs CCS	0.92	0.50-1.68	0.7781			
Hypertension	1.40	0.68-2.87	0.3645			
Diabetes mellitus	1.08	0.62-1.88	0.7830			
Hb^a^	0.82	0.62-1.08	0.1524			
Log TnT^a^	1.10	0.83-1.48	0.5045			
Log BNP^a^	1.80	1.36-2.39	<0.0001	1.70	1.22-2.37	0.0018
Log hsCRP^a^	1.21	0.91-1.61	0.1936			
Log GDF-15^a^	1.45	1.12-1.83	0.0035	1.07	0.75-1.47	0.7001
Revascularization	0.56	0.17-1.79	0.3267			
Low body weight	1.19	0.64-2.22	0.5723			
Severe CKD	2.05	1.11-3.80	0.0220	1.08	0.53-2.24	0.8261
Moderate to severe anemia	1.62	0.76-3.44	0.2127			
Heart failure	2.41	1.37-4.23	0.0022			
Anticipated use of long-term oral anticoagulation	1.10	0.52-2.33	0.8125			
PVD	3.02	1.63-5.60	0.0004	2.74	1.46-5.16	0.0018
Bleeding events and ABC-AF-bleeding score						
Low body weight	1.72	1.08-2.75	0.0229	1.13	0.68-1.85	0.6419
Severe CKD	1.80	1.07-3.02	0.0257	0.81	0.44-1.49	0.5031
Heart failure	2.72	1.74-4.28	<0.0001	1.85	1.10-3.10	0.0197
Anticipated use of long-term oral anticoagulation	1.58	0.91-2.73	0.1050	1.32	0.76-2.32	0.3244
PVD	1.43	0.77-2.64	0.2589	1.37	0.73-2.56	0.3314
ABC-AF-bleeding score^a^	1.79	1.46-2.24	<0.0001	1.61	1.25-2.06	0.0002

MACE = major adverse cardiovascular event(s); other abbreviations as in Tables 1 and 2.

a Per 1 SD increase.

**Figure 5:**
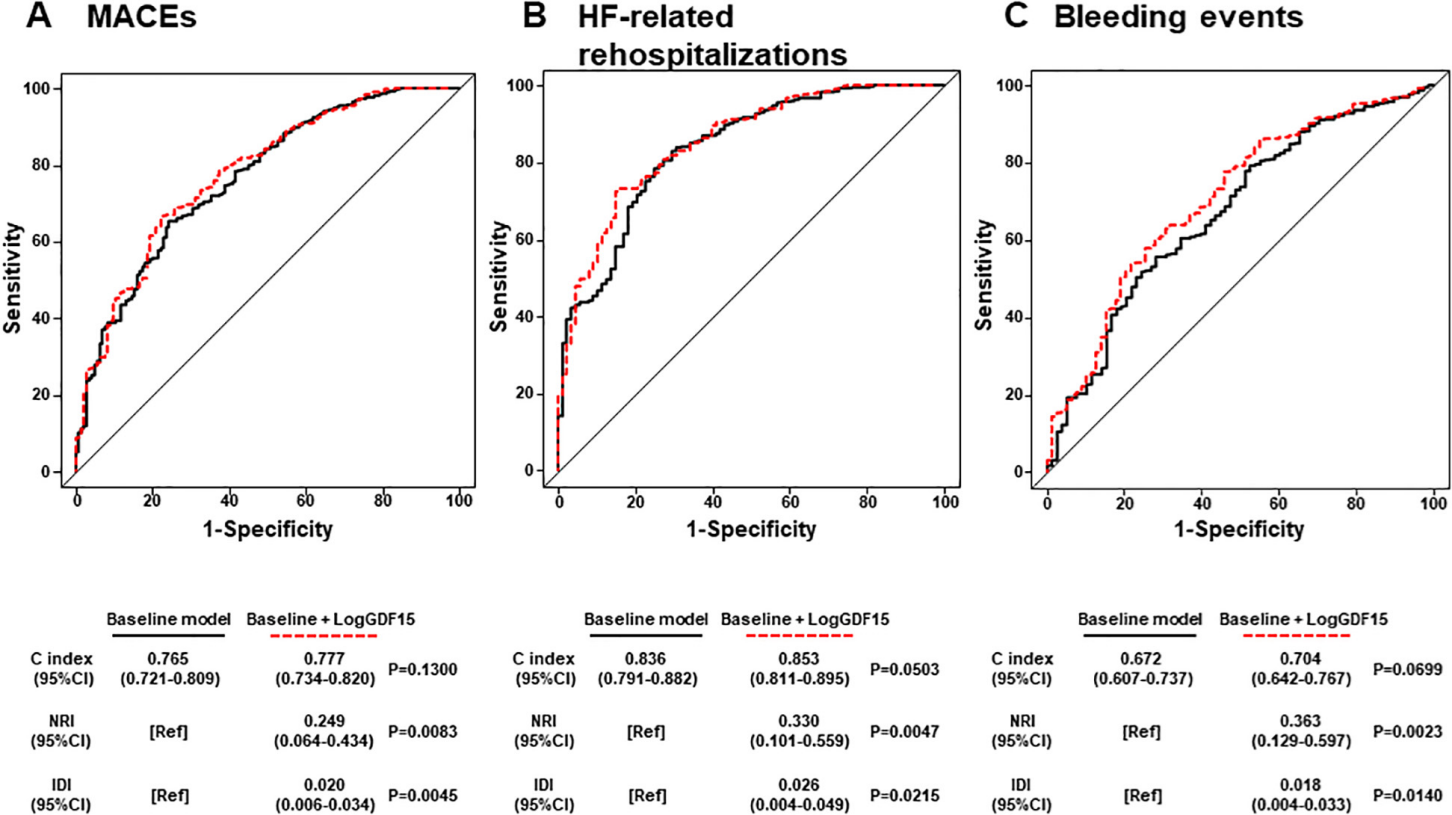
Prediction Capacity of Serum Growth Differentiation Factor-15 for Secondary Outcomes Receiver-operating characteristic curves for MACE **(A)**, HF-related rehospitalizations **(B)**, and bleeding events **(C)**. Comparison of C index, NRI, and IDI for MACE, HF-related rehospitalization, and bleeding events between baseline model with and without serum growth differentiation factor-15. The baseline model for MACE includes age, sex, hypertension, hemoglobin, troponin T (TnT), brain natriuretic peptide (BNP), and high-sensitivity C-reactive protein (hsCRP), revascularization, severe CKD, oral anticoagulation, and PVD. The baseline model for HF-related rehospitalization includes age, sex, hemoglobin, TnT, BNP, hsCRP, low body weight, severe CKD, and oral anticoagulation. The baseline model for bleeding events includes age, hemoglobin, low body weight, severe CKD, HF, oral anticoagulation, and PVD. Abbreviations as in Figures 1, 2, and 3.

To examine whether model fit and discrimination improved when serum GDF-15 was added to predictors selected in univariate Cox proportional hazards regression analysis, we evaluated improvements in the C index, NRI, and IDI for MACE and HF-related rehospitalizations. Similarly, we examined whether the addition of GDF-15 to J-HBR could improve the C index, NRI, and IDI for bleeding events. There were no significant differences in the C index for MACE, HF rehospitalization, and bleeding events between the baseline model with and without serum GDF-15. However, NRI and IDI for MACE, HF rehospitalization, and bleeding events improved significantly after adding serum GDF-15 (Figures 5A to 5C).

## Discussion

The novel findings from the present study are as follows: 1) serum GDF-15 elevation was involved in severe coronary artery disease and J-HBR criteria in patients with IHD; 2) multivariate Cox proportional hazards regression analysis demonstrated that serum GDF-15 was an independent predictor of all-cause death, MACE, HF-related rehospitalization, and bleeding events but not of thrombotic events; and 3) serum GDF-15 improved prediction ability for all-cause death, MACE, HF-related rehospitalization, and bleeding events.

### Serum GDF-15 level and HBR

The association between serum GDF-15 and major bleeding in Asian patients with IHD has not yet been examined.

Low body weight is a specific bleeding risk factor in Japanese patients with IHD.^6^ An experimental study has indicated that GDF-15 regulates appetite and body weight by binding to the glial cell–derived neurotrophic factor receptor alpha-like, expressed mainly in postrema neurons.^21^ A study comparing serum GDF-15 levels in twin pairs demonstrated a relationship between GDF-15 levels and weight loss in humans.^22^

The more severe the CKD, the higher the risk for bleeding events in patients with IHD.^6^ A high GDF-15 level was reportedly associated with the progression and incidence of CKD and mortality in patients undergoing hemodialysis.^23^, ^24^, ^25^

Anemia involved in renal dysfunction and chronic inflammation is reportedly associated with high GDF-15 levels, while iron deficiency anemia is not.^26^^,^^27^ An inverse correlation between serum GDF-15 level and hemoglobin has been reported in patients with HF and in heart allograft recipients.^28^

HF is reportedly associated with elevated circulating GDF-15 levels.^29^ Previous reports have demonstrated a significant association of high GDF-15 with mortality and HF rehospitalization in patients with HF.^30^

In the present study, AF was the primary reason for the anticipated use of long-term oral anticoagulation. It has been reported that plasma and atrial tissue expression of GDF-15 are higher in patients with AF than in those with sinus rhythm.^31^ GDF-15 is also related to clinical outcomes, including mortality, major bleeding, and stroke, in patients with AF.^14^

PVD is a common comorbidity in patients with IHD. De Haan et al^32^ demonstrated that serum GDF-15 level was correlated with the severity of PVD and was significantly associated with major amputation and all-cause mortality in patients with PVD.

We found that serum GDF-15 levels increased with increasing HBR risk, suggesting that serum GDF-15 levels, in part, reflect the accumulation of HBRs. As the significant association between serum GDF-15 and bleeding events was maintained in the multivariate analysis after adjustment for J-HBR, another mechanism should explain the close association between serum GDF-15 level and bleeding events. An experimental study demonstrated that GDF-15 could prevent platelet aggregation by inhibiting the activation of glycoprotein IIb/IIIa,^33^ which is the most abundant receptor in human platelets and acts as a binding site for fibrinogen and von Willebrand factor, resulting in interaction with coagulation factors and other platelets.^34^ Clinical studies have suggested that GDF-15 could be a marker for bleeding events in patients with IHD.^35^ Importantly, we showed that the prediction model for bleeding events was significantly improved after adding GDF-15, as evidenced by an improvement in the NRI and IDI. This result confirmed the clinical importance of GDF-15 in bleeding events and raised the possibility that GDF-15 could be a clinical factor in addition to J-HBR in patients with IHD.

### Serum GDF-15 level and clinical outcomes

Accumulating evidence indicates that GDF-15 predicts mortality and HF development.^30^ Similarly, we demonstrated for the first time that serum GDF-15 could identify and risk-stratify Japanese patients with IHD at high risk for all-cause mortality, MACE, and HF-related rehospitalization.

Despite the close relationship between serum GDF-15 levels and severe coronary artery disease, GDF-15 was not significantly associated with thrombotic events. One report showed that GDF-15 could predict thrombotic events.^35^^,^^36^ Others have shown no significant association between GDF-15 level and thrombotic events in patients with AF.^14^^,^^37^ A lower incidence of thrombotic events among Asian patients with IHD may yield this result.^5^ Therefore, thrombotic events were not the major cause of death in the high GDF-15 group. A recent study indicated the importance of bleeding events in all-cause mortality.^1^ Thus, we focused on the association of serum GDF-15 levels with major J-HBR criteria and bleeding events. As mentioned previously, serum GDF-15 levels were related to the clinical outcomes of CKD, HF, AF, and PVD.^14^^,^^24^^,^^30^^,^^32^ Therefore, the high mortality observed in the highest GDF-15 tertile group may be explained by exacerbation of HBR and bleeding events. Importantly, we showed that the prediction models for all-cause mortality, MACE, and HF-related rehospitalization improved significantly after adding serum GDF-15 levels, as evidenced by improvements in NRI and IDI. This result confirmed the clinical importance of GDF-15 in clinical outcomes and raises the possibility that GDF-15 could be an additional clinical factor to consider in patients with IHD.

### Study limitations

First, as this was a prospective observational study, we could not determine the causal relationship between serum GDF-15 levels and clinical outcomes. To eliminate the effect of GDF-15 measurement on antithrombotic medicine, optimization of medical therapy was performed by physicians who were blinded to GDF-15 value throughout the study period. Second, frailty, which is also a major criterion for J-HBR, was not assessed. Third, because cardiologists prescribed antiplatelet medication according to the guideline recommendations, the intensity and duration of antiplatelet therapy were altered during the study period. This may have affected the bleeding event rate. Finally, the optimal cutoff value for serum GDF-15 remains unclear.

## Conclusions

Our study revealed a close association among serum GDF-15 level, J-HBR, and bleeding events in Japanese patients with IHD. Serum GDF-15 level improved the prediction capacity for all-cause mortality, MACE, HF-related rehospitalization, and bleeding events. Serum GDF-15 could serve as a discriminatory factor between thrombotic and bleeding risk and is a feasible marker for major bleeding and adverse clinical outcomes in Asian patients with IHD.

Perspectives **COMPETENCY IN MEDICAL KNOWLEDGE:** We demonstrate, for the first time, the prognostic usefulness of serum GDF-15 for mortality, MACE (except for thrombotic events), HF development, and major bleeding events in Asian patients with IHD. This knowledge serves as a management tool for patients with HBR and early identification and risk stratification of high-risk patients for poor clinical outcomes.

**TRANSLATIONAL OUTLOOK:** Future study is required to confirm the feasibility and clinical utility of GDF-15 in patients with IHD across different geographic areas.

## Funding Support and Author Disclosures

The authors have reported that they have no relationships relevant to the contents of this paper to disclose.
